# Repair of recurrent rectocele with posterior colporrhaphy or non-absorbable polypropylene mesh—patient-reported outcomes at 1-year follow-up

**DOI:** 10.1007/s00192-018-03856-y

**Published:** 2019-01-09

**Authors:** Emil Nüssler, Gabriel Granåsen, Emil Karl Nüssler, Marie Bixo, Mats Löfgren

**Affiliations:** Department of Clinical Science, Obstetrics and Gynaecology, Umeå University, 90187 Umeå, Sweden

**Keywords:** Rectocele, Colporrhaphy, Non-absorbable mesh, National register data, Patient-reported outcome

## Abstract

**Introduction and hypothesis:**

The aim of this study was to compare the results of repair of isolated, recurrent, posterior vaginal wall prolapse using standard posterior colporrhaphy versus non-absorbable polypropylene mesh in a routine health care setting.

**Methods:**

This cohort study was based on prospectively collected data from the Swedish National Register for Gynaecological Surgery. All patients operated for recurrent, posterior vaginal wall prolapse in Sweden between 1 January 2006 and 30 October 2016 were included. A total of 433 women underwent posterior colporrhaphy, and 193 were operated using non-absorbable mesh. Data up to 1 year were collected.

**Results:**

The 1-year patient-reported cure rate was higher for the mesh group compared with the colporrhaphy group, with an odds ratio (OR) of 2.06 [95% confidence interval (CI) 1.03–4.35], corresponding to a number needed to treat of 9.7. Patient satisfaction (OR = 2.38; CI 1.2–4.97) and improvement (OR = 2.13; CI 1.02–3.82) were higher in the mesh group. However, minor surgeon-reported complications were more frequent with mesh (OR = 2.74; CI 1.51–5.01). Patient-reported complications and re-operations within 12 months were comparable in the two groups.

**Conclusions:**

For patients with isolated rectocele relapse, mesh reinforcement enhances the likelihood of success compared with colporrhaphy at 1-year follow-up. Also, in our study, mesh repair was associated with greater patient satisfaction and improvement of symptoms, but an increase in minor complications. Our study indicates that the benefits of mesh reinforcement may outweigh the risks of this procedure for women with isolated recurrent posterior prolapse.

## Introduction

Pelvic organ prolapse (POP) is a common condition, and women have an approximately 12–19% lifetime risk of undergoing an operation for POP [1, 2]. Unfortunately, high rates of recurrence have been reported after standard vaginal prolapse repair procedures [3, 4]. In an effort to improve surgical outcomes, mesh materials have been introduced in vaginal repair procedures. Few studies conducted to date report outcomes of the repair of an isolated POP compartment; thus far, studies have tended to include patients with prolapse in different compartments, and multiple compartments, and/or with a mixture of both recurring and primary prolapse as well as other concurrent operations (such as incontinence procedures) [3, 5].

To address this lack of information, our research team conducted a number of register-based studies comparing the use of synthetic mesh with non-mesh procedures in primary or recurrent POP patients. These studies have concerned primary cystocele [6], primary rectocele [7] and recurrent cystocele [8], with no concurrent surgery. The present, fourth, study concerns recurrent rectocele. Patients with relapse of an isolated rectocele, with no concurrent surgery, complications or medical issues, were analysed.

This study uses Patient-Reported Outcome Measures (PROMs) to measure outcomes. The rationale is that POP surgery is performed primarily to address patient symptoms, in combination with an anatomical defect that leads to surgery. In other words, if there are no patient symptoms of bulging or discomfort, an anatomical defect would not necessarily lead to surgery. Patient-reported outcomes are highly relevant and reliable and are used as the main outcome measures in this study.

## Aims

The aim was to investigate patient-reported outcomes and medical complications in these recurring rectocele patients when treated with polypropylene mesh versus native tissue repair.

## Materials and methods

This is a register-based study of patients in Sweden operated on solely for recurrent posterior vaginal wall prolapse from 1 January 2006 to 30 October 2016. Data were collected prospectively from GynOp, and the methodology was similar to that in our previous three studies concerning other vaginal compartments [6–8]. The completeness of inclusion in GynOp has continuously matched that in the National Swedish Patient Register, where all surgical procedures in Sweden are recorded by law [9]. The national quality registers, such as GynOp, operate on the basis of opt out, where patients are automatically registered unless actively declining registration [10]. From 2006 to 2010, the number of clinics reporting to GynOp increased from 46 of 62. Since 2010, 61 (99%) Swedish clinics have been reporting to GynOp.

All patients included in this study had posterior colporrhaphy or implantation of a synthetic, non-absorbable polypropylene mesh. No other compartments were operated on, nor were other operations of any type performed in any of the 626 selected patients. This was confirmed by the surgeons as part of the registration process.

Only healthy patients [American Society of Anaesthesiologists (ASA) physical status classification system I or II] who had been operated on for recurrence in posterior vaginal wall prolapse were included in this study. Patients were excluded if they had a prolapse in any other vaginal compartment, had undergone a concurrent operation of any kind (including incontinence procedures) or had been operated on using a biological mesh. Data on all patients were included in the GynOp register from prior to the operation until 1 year post-operatively.

Patients were followed up at 2 months and again at 1 year post-operatively with the internally validated questionnaires from the GynOp register, with adaptions from previously validated questionnaires [11]. Validations are done internally. Peri-operative data were registered by the surgeon. The surgeon also reviewed the patient questionnaires and conducted a surgical evaluation at 2 months and 1 year post-operatively. The surgeon’s evaluation at 2 months and at the 1-year follow up was mainly based on the patient questionnaire. Surgeons actively used the questionnaire results and scheduled extra follow-up visits or evaluations only where necessary.

A more detailed outline of the data collection process has been published by us previously [6, 12].

Continuous data were analysed without any adjustment, exactly as extracted from the register; however, but for the categorical data, the GynOp register had either binary questions or used a 5-point evaluation form (from worst outcome to best outcome). For the latter, we chose to dichotomise categorical data into binary parameters, with the best and second best outcomes as a positive outcome and the remaining three outcomes as a negative outcome.

In recognition of the importance of patients’ own evaluation of treatment results, patient-reported outcome measures (PROMs) have been developed and used more and more often for the assessment of treatment results [13]. GynOp (http://www.gynop.org) has collected extensive PROM data since 2006.

### Outcomes

We analysed both PROMs and surgeon-reported outcomes from the database. Patient-reported pain and post-operative complications were derived from the patient questionnaire at 2 months post-operation. Information about patient satisfaction, functional parameters and feeling of protrusion was extracted from the 1-year questionnaire. Improvement was defined as the patient's subjective assessment of a better wellbeing at the time of the questionnaire compared with before they were operated for POP. Satisfaction was a subjective question and shows if the operative results matched the patient's expectations.

Organ damage was reported by the surgeon either during the operation or at patient discharge, or in connection with the evaluation at 2 months post-surgery. All medical complications, including repeated operations, were registered by the surgeon, at the very latest in connection with the surgeon’s 1-year evaluation.

### Statistical analysis

We used the chi-square test for analysis of categorical data and Student’s *t* test and the Mann-Whitney U-test for continuous data. Multiple logistic regression models were constructed to examine the association between the type of operation and each of the outcomes. Risks are presented as unadjusted and adjusted odds ratios (ORs) with 95% confidence intervals (CIs) and as unadjusted risk differences (RDs). As potential confounders, we included age (continuous), pre-operative use of oestrogen (yes/no) and degree of prolapse (cm from hymen: −3 cm, > − 3 cm to < −1 cm, ≥ − 1 cm to ≤ 1 cm, > 1 cm). We used SPSS version 20.0 (SPSS Inc., Chicago, IL, USA) and Stata 12 (StataCorp, College Station, TX, USA) for the data analysis.

#### Ethics

This study and our use of data from the GynOp register have been approved by the Regional Ethical Review Board in Umeå, Sweden (Dnr 08-076 M).

## Results

Of a total of 51,095 POP operations registered during the study period, 626 (1.2%) met the strict inclusion criteria. The 626 patients (433 women who underwent classic posterior colporrhaphy and 193 operated on using a non-absorbable mesh) included in our material were consecutively enrolled in GynOp. Table 1 compares the baseline characteristics of the patients in the two groups. Women in the mesh group were significantly older and more commonly had severe prolapse compared with the women in the colporrhaphy group. Therefore, age and degree of prolapse were adjusted for in the statistical analysis.

**Table 1:** Characteristics of participants with recurrent posterior vaginal wall prolapse receiving classic posterior colporrhaphy or mesh implants, Sweden, 2006–2016

	Implant (*N* = 193)		No implant (*N* = 433)		*p* value
Mean age, years (SD)	63.8 (10.4)		58.6 (12.5)		< 0.001
Patient questionnaires					
BMI (SD)	26.7 (3.7)		26.6 (4)		0.735
	*n*	(%)	*n*	(%)	
Parity					
0–2	84	(52.8%)	171	(52.3%)	0.989
3+	75	(47.2%)	156	(47.7%)	0.989
Missing	34	(17.6%)	106	(24.5%)	0.072
Smoking					
Yes	8	(5.1%)	25	(9%)	0.198
No	149	(94.9%)	253	(91%)	0.198
Missing	36	(18.7%)	155	(35.8%)	< 0.001
Pre-operative oestrogen					
Yes	29	(16%)	42	(9.7%)	0.071
No	164	(84%)	391	(90.3%)	0.071
Missing	0		0		
Surgeon-completed forms					
Degree of prolapse^a^					
0 (−3 cm)	6	(4.5%)	4	(1.7%)	0.201
1 (> − 3 cm to < −1 cm)	10	(7.6%)	18	(7.6%)	1
2 (≥ − 1 cm to ≤ 1 cm)	80	(60.6%)	183	(77.5%)	< 0.001
3/4 (> 1 cm)	36	(27.3%)	31	(13.1%)	0.001
Missing	61	(31.6%)	197	(45.5%)	0.002

*BMI* body mass index, *SD* standard deviation, *N* total number of patients, *n* number of patients

^a^Degree of prolapse (± cm from hymen)

Patient satisfaction, patient-reported complications, pain and patient-assessed urogynaecological parameters are shown in Tables 2 and 3. Patients were considered cured if they never or hardly ever had a feeling of genital protrusion at 1 year after the operation. The subjective cure rate for the mesh group was higher compared with the posterior colporrhaphy group (OR = 2.06; 95% CI 1.03–4.35), corresponding to a number needed to treat (NNT) of 9.7. In addition, the mesh group were generally more satisfied and reported a higher degree of improvement overall at 1 year post-operation. There was no statistical difference in patient-reported complications, re-admissions due to complications, urinary infection, urinary retention or post-operative pelvic pain between the two groups. The rate of self-administered painkillers reported by the patients was not significantly different between the two groups (*p* = 0.123).

**Table 2:** Patient-reported outcomes: recurrent posterior wall prolapse repaired using classic posterior colporrhaphy or a mesh implant. Sweden, 2006–2016

Patient satisfaction							
	N	Missing (%)	RD	OR_u_^a^	(95% CI)	OR_Ab_	(95% CI)
Satisfaction at 1 year							
No implant	433	155 (35.7%)		1			
Implant	193	66 (34.1%)	13.9%	1.96	(1.22–3.21)	2.38	(1.2–4.97)
Improvement at 1 year							
No implant	433	202 (46%)		1			
Implant	193	65 (33.6%)	16.1%	2.41	(1.43–4.19)	2.13	(1.02–3.82)
Patient-reported complications							
	N		RD	OR_u_^a^	(95% CI)	OR_Ab_	(95% CI)
Patient-reported complications within 8 weeks, with medical attention sought							
No implant	433	101 (23.3%)		1			
Implant	193	27 (13.9%)	1.2%	1.07	(0.68–1.68)	1.23	(0.58–1.99)
Patient-reported complications, after 8 weeks and within 1 year. Receiving medical attention (only patients not previously reported)							
No implant	433	147 (23.3%)		1			
Implant	193	66 (34.2%)	−0.5%	0.97	(0.56–1.67)	0.81	(0.38–1.68)
Complications needing hospitalisation of patient up to 8 weeks after surgery							
No implant	111	43 (30.6%)		1			
Implant	61	(34.4%)	5.81%	1.8	(0.63–5.22)	2.15	(0.6–7.87)
Urinary infection post-operatively							
No implant	433	4 (0.09%)		1			
Implant	193	6 (3,1%)	N/A	N/A			
Urinary retention (more than 1 day post-operatively up to 8 weeks)							
No implant	433	71 (16.4%)		1			
Implant	193	17 (8.8%)	1.46%	1.79	(0.57–5.47)	0.99	(0.19–4.31)
Patient-reported pain							
	*N*		Median	(SD)	Median	[25%. 75%]	*p* value
Number of days using painkillers at home after surgery							
No implant	433	197 (45,5%)	6.8	(7.55)	5.0	[2. 10]	
Implant	193	56 (29.0%)	5.7	(6.08)	4.0	[2. 7]	0.123
	N		RD	OR_u_^a^	(95% CI)	OR_Ab_	(95% CI)
Pelvic pain (within 8 weeks)							
No implant	433	104 (24,0%)		1			
Implant	193	31 (16.1%)	1.26%	1.71	(0.49–5.78)	3.36	(0.77–17.39)
Patient-reported cure rate							
Patient-reported absence of genital protrusion 1 year after surgery							
No implant	433	158 (36.5%)					
Implant	193	71 (36.8%)	10.3%	1.80	(1.07–3.12)	2.06	(1.03–4.35)

*N* Number of patients eligible to answer a specific question, *CI* confidence interval, *N/A* not applicable, *OR* odds ratio, *RD* risk difference, *SD* standard deviation

^a^Unadjusted

^b^Adjusted for age, pre-operative oestrogen and degree of prolapse

**Table 3:** Patient-reported functional parameters: recurrent posterior wall prolapse repaired using classic posterior colporrhaphy or a mesh implant, Sweden, 2006–2016

Functional parameters								
	*N*	Missing (%)		RD	OR_u_*	(95% CI)	OR_A†_	(95% CI)
Started having sexual intercourse (patients who did not engage in intercourse before the operation)								
No implant	433	208 (48%)					1	
Implant	193	89 (46%)		−1.8%	0.75	(0.26–1.87)	1.47	(0.4–4.96)
Stopped having sexual intercourse (patients who reported engaging in intercourse before surgery)								
No implant	433	208 (48%)					1	
Implant	193	89 (46%)		5.2%	2.10	(0.88–4.96)	1.10	(0.33–3.43)
Dyspareunia, improved or symptom-free								
No implant	225	140 (62.2%)					1	
Implant	104	69 (66.3%)		4.71%	1.38	(0.48–3.76)	1.35	(0.16–8.52)
Dyspareunia, worsened								
No implant	225	140 (62.2%)					1	
Implant	104	69 (66.3%)		−1.51%	0.87	(0.23–2.76)	2.04	(0.36–9.93)
Dyspareunia, de novo								
No implant	225	140 (62.2%)					1	
Implant	104	69 (66.3%)		−4.2%	0.39	(0.02–2.39)	0.71	(0.03–5.78)
Urinary incontinence, improved								
No implant	433	205 (47,3%)					1	
Implant	193	83 (43.0%)		−1.8%	0.91	(0.53–1.53)	0.80	(0.40–1.57)
Urinary incontinence, worsened								
No implant	433	205 (47,3%)					1	
Implant	193	83 (43.0%)		−3.16%	0.73	(0.34–1.48)	0.98	(0.31–2.77)
Urinary incontinence, de novo								
No implant	433	205 (47,3%)					1	
Implant	193	83 (43.0%)		5.9%	1.62	(0.85–3.07)	1.76	(0.73–4.23)
Urge problems, improved								
No implant	433	212 (48.9%)					1	
Implant	193	83 (43.0%)		1.2%	0.94	(0.56–1.55)	0.88	(0.45–1.68)
Urge problems, worsened								
No implant	433	212 (48.9%)					1	
Implant	193	83 (43.0%)		−5.39%	0.57	(0.25–1.2)	0.67	(0.22–1.80)
Urge problems, de novo								
No implant	433	212 (48.9%)					1	
Implant	193	83 (43.0%)		0.51%	1.05	(0.5–2.11)	1.21	(0.44–3.13)
Defecation problems, improved								
No implant	433	201 (46.4%)					1	
Implant	193	77 (39.8%)		2.59%	1.11	(0.71–1.74)	0.86	(0.48–1.54)
Defecation problems, worsened								
No implant	433	201 (46.4%)					1	
Implant	193	77 (39.8%)		1.29%	1.25	(0.48–3.05)	0.83	(0.2–2.88)
Defecation problems, de novo								
No implant	433	201 (46.4%)						
Implant	193	77 (39.8%)		4.31%	1.8	(0.79–4.03)	1.98	(0.61–6.68)
Return to ADLs^a^								
	*N*	Mean	missing	(SD)	Median	[25%, 75%]	*p* value	
No implant	433	5.1		5.1	3	[1, 7]		
Implant	193	4.9		4.6	3	[1, 7]	0.966	

*Unadjusted. †Adjusted for age, pre-operative oestrogen and degree of prolapse. N = Number of patients eligible to answer a specific question. CI = confidence interval; N/A = not applicable; OR = odds ratio; RD = risk difference; SD = standard deviation

Table 3 summarises the patient-reported changes in functional parameters. Overall, there seemed to be no discernible difference in functional urogynaecological effect of the operation between the two groups. Changes in sexual behaviour (proportions of patients who continued, stopped or started having penetrating sex after the operation) and in degree of dyspareunia were similar in both groups, as was the time to return to normal daily life. Patient-reported urinary incontinence 1 year after the operation was comparable in the two groups. De novo defecation problems, as well as patient-reported worsening of symptoms, were similar for both groups.

The use of health care resources and the medical complications recorded by the surgeon are shown in Table 4. There was a significant difference in surgeon-reported minor medical complications within 12 months in favour of colporrhaphy (OR = 2.27; 95% CI 1.77–2.91). Also, operation time and time in hospital were shorter for patients in the posterior colporrhaphy group (Table 4). There was no significant difference between the operation types regarding re-operation rates within 1 year or peri-operative bleeding. There was no other organ damage, and no recto-vaginal fistulas occurred in any of the 626 operations.

**Table 4:** Surgeon-reported parameters: recurrent posterior wall prolapse repaired using classic posterior colporrhaphy or a mesh implant, Sweden, 2006–2016

**Resource parameters**							
	N	Missing (%)	Mean	(SD)	Median	[25%, 75%]	p value
* Operation time (minutes)*							
No implant	433	51 (11.7%)	44.34	22.4	40	[30, 55]	
Implant	193	38 (19.7%)	46.88	16.6	44	[35, 57]	0.006
* Time in hospital (days)*							
No implant	433	6 (1.3%)	0.71	1	0	[0, 1]	
Implant	193	8 (4.1%)	1	0.9	1	[1]	< 0.001
**Medical complications**							
Missing	N	Missing	RD	OR_u_*	(95% CI)	OR_A†_	(95% CI)
* Surgeon-reported complications (minor) within 12 months*							
No implant	433	0					
Implant	193	0	11.71%	2.18	(1.42–3.35)	2.74	(1.51–5.01)
* Re-operation within 12 months*							
No implant	433	0					
Implant	193	0	1.15	2.27	(0.53–9.69)	4.99	(0.88–39.4)
* Haemorrhage during operation (ml)*							
	N	Mean (ml)	Missing (%)	(SD)	Median	[25%, 75%]	p value
No implant	433	34	0	37.1	25	[10, 50]	
Implant	193	38	0	28.3	25	[20, 50]	0.17
**Organ damage**							
* Bladder lesion*							
Missing	N	missing	RD	OR_u_*	(95% CI)	OR_A†_	(95% CI)
No implant	433	0					
Implant	193	0	N/A	N/A			
* Urethral lesion*							
No implant	433	0					
Implant	193	0	N/A	N/A			
* Intestinal lesion*							
No implant	433	0					
Implant	193	0	N/A	N/A			
* Vaginal lesion*							
No implant	433	0					
Implant	193	0	N/A	N/A			
* Fistula*							
No implant	433	0					
Implant	193	0	N/A	N/A			

*Unadjusted

†Adjusted for age, pre-operative oestrogen and degree of prolapse

N = Number of patients eligible to answer a specific question

CI = confidence interval

N/A = not applicable, OR = odds ratio, RD = risk difference, SD = standard deviation

## Discussion

This register-based study of 626 operations for isolated, recurring rectocele shows that the benefits of using non-absorbable mesh reinforcement may outweigh the risks and indicates that permanent surgical mesh may be a viable treatment option for this particular patient group, if confirmed by longer term studies. In this article, we provide information from real-life routine health care settings that might be helpful both to the surgeon and for the patient in the decision regarding which process will be most beneficial for the patient.

Surgical mesh has been used in POP surgery for nearly a decade, but there is still no clear international consensus on how, when and with which type of prolapse it might be advantageous to use such implants. The surgeon must make a risk assessment for each individual case, and the patient must be able to make a personal, informed decision as to whether she wants an augmentation using an implant. Essential in this process is a clear, realistic description of both the desired and unwanted effects of the planned surgery.

The evaluations of mesh use in prolapse surgery are often unstratified, where the individual vaginal compartments are either pooled or unspecified, or assumed to be of equal impact, and this produces ambiguous results regarding site-specific evaluations of impact [3, 14]. From continuous monitoring of GynOp register items, we know that the posterior vaginal wall is the second most common site of POP. In Sweden, two-thirds of the total number of synthetic mesh operations in POP are performed on recurrent patients, and this group is therefore of particular interest.

As in previous articles published by us in collaboration with the GynOp register [6–8], our material consisted of a very specific patient subgroup within POP. This was done to achieve a compartment-specific, clinically applicable evaluation of mesh reinforcement in the main individual patient groups within the POP group. Even though our work encompasses a highly specific patient group (patients operated solely for recurring rectocele), the sample size is large because of the extensive material provided by the register, making it possible to analyse subgroups without compromising the precision of the results.

The study has varying sample sizes for different parameters. This variation is due not to missing patients, but to some patients not being eligible to answer the questions. For example, in Table 2 only patients who had previously reported a complication within 8 weeks received the question of whether these complications had led to hospitalisation. Similarly, there are more 8-week results available than 1-year results, simply because not all the patients who answered the first questionnaire were eligible to answer the 1-year questionnaire at the date of data extraction from the register.

Some parameters had a high degree of missing information, such as the degree of prolapse and smoking status (Table 1), and both may influence severity and recurrence. However, none of these instances were such that statistical analysis was not feasible.

There was a difference between patient groups regarding both age and degree of prolapse. Both could affect the outcome of the operation and therefore represent possible confounding factors in our study. We measured the position of the anterior respectively posterior vaginal wall prolapse in relation to the hymen in cm for each group. Statistically significant differences can be seen between groups 2 and 3 (the second most and most severe prolapse groups). In relative terms there are 17% more native patients in group 2 and 14% more mesh patients in group 3.

This would potentially diminish cure rates for the mesh group; thus, our conclusion about mesh benefits may be underestimated.

The mean age difference between groups was 4.8 years. Whether or not this age difference will affect the outcome of the operation and patient satisfaction is unknown.

In the literature, concerns have been expressed that implants might increase urinary incontinence. In our material, we show that in a routine health care setting there was no difference in patient-reported de novo incontinence and that existing urinary incontinence was actually improved equally for both patient groups.

The only symptom specific to prolapse is the awareness of a vaginal bulge or protrusion [15], and this is regarded as a valid way of measuring the existence of prolapse [16–19]. Other more specific urogynaecological symptoms have been shown to have a very weak link with direct measures such as the Pelvic Organ Prolapse Quantification (POPQ) tool compared with the symptom of “bulging” [19]. Therefore, we regard the patient-reported cure rate as the most important clinical outcome measurement.

Patient-reported cure rates have the inherent problem of not having been objectively verified by a physician.

De novo prolapse in a new compartment, therefore, would be reported as a failed operation even though it might be unrelated to the surgical procedure. This might overestimate the total amount of failure, but it would not influence the differences between groups. Additionally, as the patients’ self-reported lack of bulging symptoms and their wellbeing are the goal of the operation, it is our belief that the anatomical evaluation is secondary.

More patients who were treated with mesh reinforcement compared with patients undergoing classic posterior colporrhaphy were cured at 1 year post-operation, with an NNT of 9.7. In addition, mesh reinforcement was superior or equal to native tissue repair in all parameters except for the number of post-operative surgeon-reported complications within 1 year. The number needed to harm (NNH) for any type of surgeon-reported complication was 8.5. This, if analysed in more practical terms, means that, for every 100 implants used on this particular patient group instead of native tissue repair, 10.3 additional patients will be cured, but simultaneously an additional 11.7 cases of surgeon-reported complications will occur. We reported only “minor surgical complications” because no major post-operative surgical problems occurred more frequently in either of the two homogeneous groups, and overall such major complications, including mesh erosions, were rare.

The strengths of this study are the large sample size, the fact that it mirrors results from “real life” and that it shows effectiveness rather than efficacy. The high rate of inclusion in GynOp means that the yearly registration of operations in GynOp represents the total number of operations performed in Sweden. Samples from GynOp are therefore complete national materials. The patient participation rate was very high for both the 2-month and the 1-year follow-up questionnaires (95% and 85%, respectively). This high inclusion rate has been attributed to the ubiquitous personal Scandinavian social security number and universal public health care coupled with a high degree of trust in the public authorities.

The main limitation of this study is the possibility of bias. While selection bias is likely to be a minor problem, as suggested above, information bias and confounding might still exist. Our mesh group had a greater degree of prolapse compared with the colporrhaphy group, but this selection bias probably did not affect the outcomes in favour of the mesh group. Some information or recall bias is likely because data are aggregated from patient questionnaires. In the register questionnaire, we asked the patients for the number of days they had taken painkillers at home. This parameter may be biased by different instructions from different clinics and may reflect clinical routines more than actual patient pain.

Further research in the form of a randomised trial would be needed to confirm with certainty any causality found in this cohort study. However, as this specific patient group encompass roughly 1.2% of all Swedish prolapse operations, this would require a randomisation of every single eligible Swedish patient for at least 5 years to get more than 150 patients per group (and, therefore, sufficient analytical power). A trial of this magnitude would be nonsensical, partly because of the hugely complicated process and partly because of the very small group of patients who would gain from the results. This probably also explains the small number of publications on compartment-specific surgery [20] and the almost complete lack of literature regarding recurrent rectocele operations alone.

Complication registration within the database is not implant-specific, but is designed to cover all types of expected surgical complications. Therefore, we cannot distinguish between complications characteristic of implants (e.g. bleeding due to mesh erosions) and general surgical complications (e.g. bleeding from the vaginal suture line). Similarly, regarding our registration of patient-reported complications, the severity of the reported complication is not graded. The criterion for the event of a “patient-reported complication” is that the event led to further unscheduled contact with the health care provider.

In our study, a bias is possible if clinical factors influence the decision whether or not to use mesh. Recently published data by GynOp show that the clinical factors such as age, BMI, etc., have no influence on the decision whether or not to use mesh in daily practice and that the clinical practice based on these clinical factors varies widely throughout the country. An abiding bias is therefore unlikely in our opinion [21].

Mesh types are not all alike and, therefore, as we utilise all kinds of non-resorbable polypropylene mesh, the subtle differences between brands would be overlooked. However, there is no difference in the general method of application of these mesh types or regarding the extent of the operation between brands, and all meshes in this analysis were made solely from polypropylene, generalising the results.

This is the fourth study based on GynOp that analyses operative outcome following the most common types of POP with or without mesh in the anterior or posterior compartments. In the three previous, methodologically similar articles, mesh had a superior 1-year cure rate but other disadvantages and drawbacks. In the present study, our overall conclusion for recurrent rectocele is that mesh implants are more beneficial than posterior colporrhaphy. In primary posterior vaginal wall repair, and also in both primary and recurrent anterior vaginal wall repair, the overall circumstances regarding the advantages of mesh use are more complex. A similar complex picture of the benefits of mesh augmentation can be found in a recently published randomised controlled trial [5].

## Conclusion

For patients with isolated rectocele relapse, mesh reinforcement enhances the likelihood of success compared with colporrhaphy at 1-year follow-up. Also, in our study, mesh repair was associated with greater patient satisfaction and improvement of symptoms, but an increase in minor complications. Our study indicates that the benefits of mesh reinforcement may outweigh the risks of this procedure for women with isolated recurrent posterior prolapse.
